# Efficacy and Safety of Thermal Ablation for Treating Lymph Node Metastasis From Papillary Thyroid Carcinoma: A Systematic Review and Meta-Analysis

**DOI:** 10.3389/fonc.2022.738299

**Published:** 2022-04-01

**Authors:** Zheng Ding, Juan Chen, Zhiguang Chen, Xiaoke Zeng, Pengchao Zheng, Xuemei Wang, Xinwu Cui, Liang Sang

**Affiliations:** ^1^ The First Affiliated Hospital of China Medical University, Shenyang, China; ^2^ Department of Ultrasound, The First Hospital of China Medical University, Shenyang, China; ^3^ Departmant of Medical Ultrasound, Tongji Hospital, Tongji Medical College, Huazhong University of Science and Technology, Wuhan, China

**Keywords:** thermal ablation, radiofrequency ablation, microwave ablation, laser ablation, lymph node metastasis, papillary thyroid carcinoma

## Abstract

**Objective:**

To evaluate the efficacy and safety of thermal ablation, including radiofrequency ablation (RFA), microwave ablation (MVA), and laser ablation (LA), for treating lymph node metastasis (LNM) from papillary thyroid carcinoma (PTC).

**Design and Methods:**

PubMed and EMBASE were searched for studies reporting the efficacy and safety of thermal ablation for treating LNM in PTC. After selecting the relevant literature (including 11 papers, 208 patients, 412 lymph nodes), the QUADAS-2 tool was used to evaluate its quality. Then, both the fixed-effects and random-effects models combined with subgroup analysis were used to calculate data on volume changes in metastatic lymph nodes and changes in serum thyroglobulin (Tg) levels. We pooled the proportion of major and overall complication rates and complete disappearance rates and used subgroup forest plots and funnel plots for visual representation. Because of publication bias, we also performed a trim-and-filled model for correction. The rate of recurrence and distant metastasis with ablated details were pooled.

**Results:**

In the 11 articles (208 patients and 412 diseased lymph nodes), all thermal ablation methods showed effectiveness in reducing lymph node volume (P = 0.02) and serum Tg levels (P < 0.01) which showed no between-group difference. The pooled proportion of major complications was 0%(95% CI: -0.14; 0.15, P = 1) and the overall complication rate was 5% (95% CI: -0.09; 0.20, P = 1), which revealed no significant difference among modalities. The pooled proportion of the complete disappearance rate was 82% (95% CI: 0.43; 0.96, P < 0.01) and the data with statistical significance which contains RFA and LA showed complete disappearance rate was 59% and 81% respectively.

**Conclusion:**

All thermal ablation methods, including RFA, MWA, and LA, were effective and safe for treating LNM in PTC and were especially suitable for nonsurgical patients. Besides, subgroup analysis showed no significant difference, except for LA is better than RFA in complete disappearance rate.

## Introduction

Thyroid cancer has become the most common endocrine malignancy, and its incidence has increased faster than that of many other malignant tumors in many other countries in recent decades. Despite the sharp rise in the incidence, the mortality rate of thyroid cancer has remained stable. Papillary thyroid carcinoma (PTC) is the most common subtype of thyroid cancer. It is a typically indolent disease with a high cure rate; however, related lymph node metastasis (LNM) is frequently observed after resection of the PTC (1, 2). Surgery and/or radioactive iodine therapy is currently the preferred treatment, but due to many factors, a considerable proportion of patients cannot undergo surgery or radioactive iodine therapy, such as those who have had many previous surgeries, an inaccessible tumor position or small size, and poor nutritional status (3).

In many cases related to neck diseases, thermal ablation, including radiofrequency ablation (RFA), microwave ablation (MWA), and laser ablation (LA), have performed well. Complications of surgical treatment include nerve injury (the superior laryngeal nerve and recurrent laryngeal nerve), thyroid dysfunction, significant blood loss, and slow recovery. Compared to surgical treatment, thermal ablation is a relatively safe and economical treatment due to its low complication rate and cost (4–8). Therefore, it can be used to treat more patients with related diseases and be applied repeatedly instead of performing many surgeries over time to deal with newly discovered PTC lymph node metastases.

Recent studies come from many regions and hospitals that have shown the safety and effectiveness of thermal ablation for treating PTC and LNM from PTC (9–22). Our purpose is to verify the efficacy and safety of thermal ablation for patients with metastatic lymph nodes from PTC, and the results obtained through this verification can provide a certain degree of guidance for clinical practice.

## Materials and Methods

### Literature Search Strategy

We searched the PubMed and EMBASE databases to find published articles on the review topic. We used the following search terms: [(“Metastatic” AND “Lymph nodes”) AND (thyroid) AND (“carcinoma” OR “cancer” OR “malignancy”) AND (“radiofrequency ablation” OR “RFA” OR “laser ablation” OR “LA” OR “microwave ablation” OR “MWA” OR “thermal ablation”)]. The search was updated until 28 February 2021, with English language only. Then, we applied a rigorous screening of the articles. After deleting duplicates, we reviewed their abstracts to obtain relevant articles. Finally, the full-text review was carried out, and studies with sample sizes that were too small were excluded.

### Inclusion Criteria

(a) Both retrospective and prospective studies were included. (b) The study population was patients with LNM of PTC who had undergone thyroidectomy, and the diagnosis was confirmed by ultrasound-guided fine-needle aspiration biopsy (FNAB). (c) Patients received only one modality of LA, RFA, or MWA. (d) Detailed data were reported, including the number, size, and volume of affected lymph nodes at the initial diagnosis and follow-up, the rate of recurrence and distant metastasis, complications, and serum Tg level.

### Exclusion Criteria

(a) Cases that were given more than one treatment. (b) Lack of a detailed description of the lymph nodes and patient numbers, effective rate, recurrence rate, metastasis rate, and complications. (c) There was only a primary lesion without lymph node metastasis. (d) The follow-up was less than six months.

### Data Extraction

We extracted the following data from the 11 studies into the data form: (a) First author, affiliation, year of publication, study design; (b) The number, age, and sex ratio of the cases; (c) Ablation treatment, the type, size, and volume of metastatic lymph nodes, ablation time and energy, changes of lymph nodes during follow-up (1 month, 3 months, 6 months, 12 months, 18 months, and 24 months); (d) The incidence of complications, local recurrence rate, distant metastasis rate, the proportion of complete disappearance of lymph node metastasis, the average volume reduction rate, the Tg level.

Complications: There were two kinds of complications: major complications and minor complications. Major complications may lead to permanent sequelae or death, while minor complications do not cause sequelae and can be corrected in a short time (23, 24). For example, hypothyroidism and irreversible voice changes are regarded as major complications, while bleeding, fever, cough, and temporary hoarseness are minor complications.

In the included studies, “no cancer seeding”, “lymph nodes undetectable”, “complete remission”, “nodes were negative”, “completely disappeared” mean complete disappearance; “remained detectable”, “partial response”, “nodes positive”, and “scar-like” implied that they had not disappeared completely. FNAB detects local recurrence in the ablated lymph nodes’ central and marginal areas; “no regeneration” represents no local recurrence. Words such as “local control” refer to the absence of distant metastases.

### Data Synthesis and Statistical Analysis

We conducted a meta-analysis on the extracted data to obtain the average volume reduction rate and subgroup analysis of the reduction rate of Tg levels in serum at same time. Then, we analyzed and described the proportion of complete disappearance, local recurrence rate, distant metastasis rate, and incidence of complications of metastatic lymph nodes of primary PTC after the main treatment. To calculate and guarantee the objectivity of the data, the fixed-effects model and random-effects model were both adopted (25). Also, we conducted between-group difference by analyzing part with sufficient data. After that, we calculated heterogeneity using the inconsistency index I^2^ (I^2^ > 25% suggests low heterogeneity; I^2^ > 50%, moderate; and I^2^ > 75%, high) with the Q statistic and P value (26). When P > 0.05 and I^2^ < 50%, we chose the fixed-effects model; in contrast, we used the Hartung-Knapp-Sidik-Jonkman random-effects model to avoid false-positives when there was high heterogeneity and low quality of the studies, with P ≤ 0.05 and I^2^ > 50% (27–29).

For the pooled estimated outcome, we used the correlation analysis method to calculate the major complication rate and overall complication rate, as well as the complete disappearance rate, with forest plots and subgroup plots. We also drew 2 funnel plots to show the publication bias of the overall complication rate and complete disappearance rate. Considering that the number of included documents was greater than 10, we used Peter’s examination to evaluate the publication bias. After deleting invalid data, both the major complication rate and disappearance rate were corrected using the trim and fill method, and a funnel plot was used to show the publication bias (30, 31). Peter’s linear regression asymmetry test was used to test the statistical significance (32, 33) (P < 0.01 means a significant bias).

Quality assessment of the statistical analyses was calculated by RevMan 5.4 software, and other statistical analyses were calculated by R studio 1.4 (R version 3.6.2) with the “meta” and “metafor” packages.

## Results

### Literature Search

**Figure 1:**
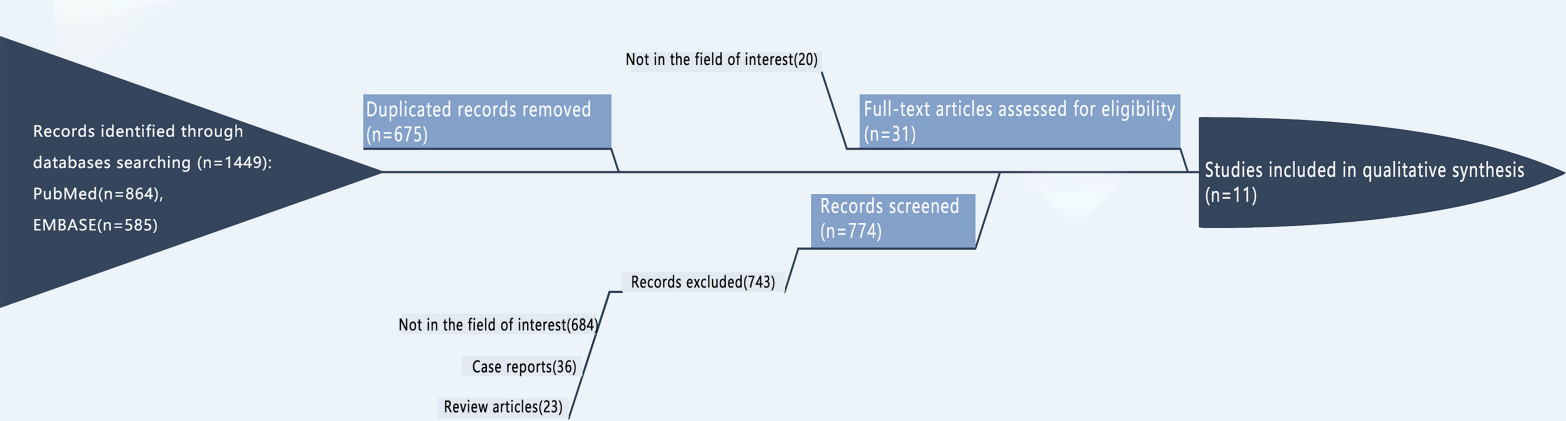
Flow diagram of study screening.

The search process is shown in **Figure 1**. We searched 1449 papers, of which 11 papers, 208 cases, and 412 lymph nodes were finally selected after applying the inclusion/exclusion criteria. After removing 675 duplicated records, we excluded 743 studies, including those not in the field of interest (684), case reports (36), and review articles (23). The full-text screening was conducted on the remaining 31 studies, and only 11 met the inclusion criteria. Although the publication date of the articles was not restricted, the selected studies were all published after 2009, with a certain degree of timeliness and adaptability.

### Literature Quality

The data were extracted by one author and verified by another to ensure accuracy and validity.

**Figure 2:**
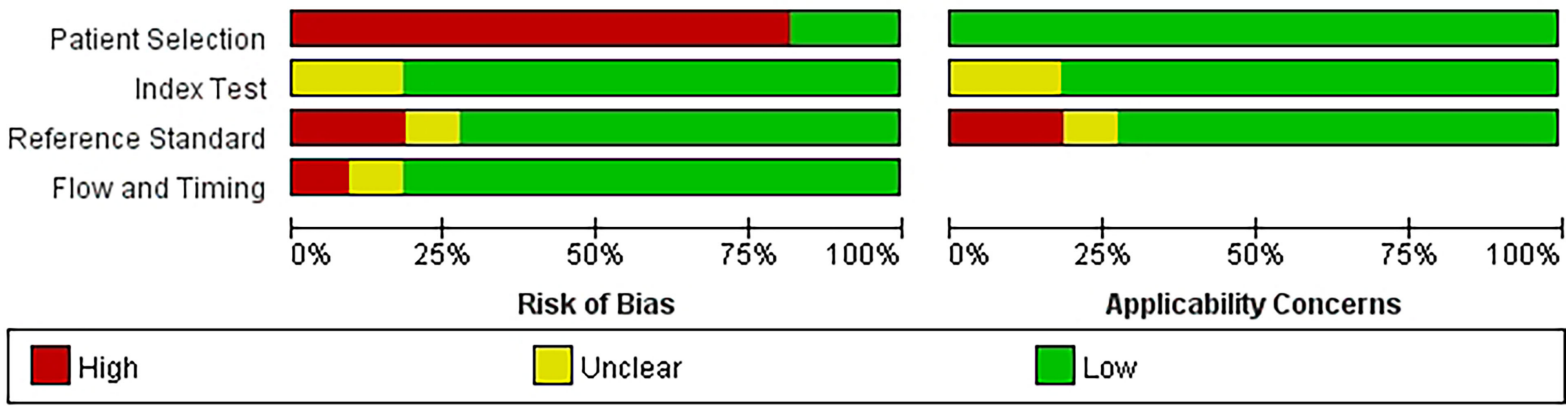
Quality Assessment of Diagnostic Accuracy Studies-2.

With the help of the built-in tailored questionnaire system and criteria provided by the Quality Assessment of Diagnostic Accuracy Studies-2 (QUADAS-2), as well as the image generation tool of Review Manager 5.4 software (34, 35), the two reviewers independently evaluated the methodological quality of the 11 included studies. The outcome is shown below (**Figure 2**).

### Characteristics of the Included Studies

The detailed features of the 11 included studies (3, 4, and 4 studies treated with RFA, MWA, and LA, respectively) are shown in **Table 1**; 9 studies were retrospective, and 2 were prospective. After a preliminary study of the demographic characteristics of the patients, we found that the mean ages of these patients ranged between 37.2 and 63.2 years, and approximately 64.0% (133/208) of the patients were women. Among the 11 studies, 3 were European (44 cases), and the remaining 8 were Asian (164 cases). The mean follow-up time after the treatment was greater than 8.4 months.

**Table 1:** Characteristics of the included studies.

First author	Affiliation (study period)	Year	Design	Type of thermal ablation	No. Patients	No. MLN	Age mean±SD	male/female	follow-up(months) mean±SD
Guang et al. (9)	Chinese PLA General Hospital,China (2013~2014)	2017	retrospective	RFA	33	54	43.7±10.7 (22~67)	11/22	21±4 (12-24)
Lim et al. (22)	Department of Radiology, Seoul Soonchunhyang University Hospital, Yongsan-Gu Seoul, Korea (2008~2012)	2015	retrospective	RFA	39	61	52.8± 16.7 (21–92)	14/25	26.4±13.7
Wang et al. (10)	Zhejiang Cancer Hospital, Hangzhou, China (2013~2014)	2014	retrospective	RFA	8	20	43.6±9.3 (30~58)	1/7	9.4±5.1 (6-20)
Zhou et al. (11)	Rui Jin Hospital of Shanghai Jiao Tong University,China (2017~2018)	2019	retrospective	MWA	14	21	45.1±12.1 (30~64)	3/11	8.4±4.1 (3-18)
Teng et al. (12)	China-Japan Union Hospital of Jilin University,China (2014~2015)	2018	retrospective	MWA	11	24	40.36±10.52 (31~59)	3/8	32 (no details)
Cao et al. (18)	China-Japan Friendship Hospital, Beijing, China (2015~2018)	2020	retrospective	MWA	14	38	46.9 ±11.9 (28-73)	3/11	23.6±9.3 (12-36)
Han et al. (19)	Chinese People's Liberation Army General Hospital (2015~2020)	2020	retrospective	MWA	37	98	43.58±13.77 (14-74)	17/20	11.09 ±9.21
Papini et al. (14)	Italian thyroid center (2009~2010)	2013	prospective	LA	5	8	53.6±18.3	1/4	12 (no details)
Mauri et al. (13)	A public hospital (2010~2012)	2013	retrospective	LA	15	24	62±14 (32~80)	8/7	12 (no details)
Mauri et al. (15)	Division of Interventional Radiology, European Institute of Oncology, Milan, Italy. (2010~2013)	2016	retrospective	LA	24	46	63.2±13.2 (32~80)	11/13	30±11 (12-45)
Guo et al.* (16)	The First Affiliated Hospital of Guangxi Medical University (2016~2017)	2020	prospective	LA	8	18	37.2±15.94 (18~72)	3/5	12 (no details)

*Paper from Guo et al. have been deleted benign nodes data.

### Changes in the Lymph Nodes Volume After LA, RFA and MWA

**Figure 3:**
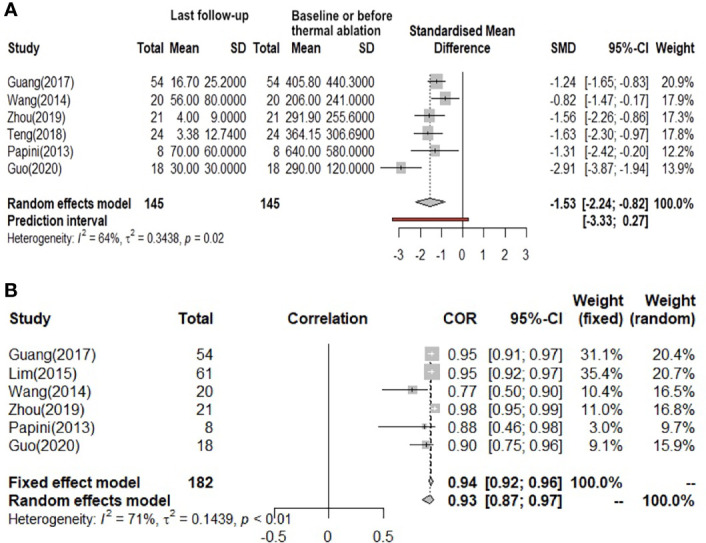
**(A)** Before and after changes in the volume of lymph nodes. **(B)** The reduction rate of lymph nodes volume.

The overall pooled estimates for the standardized mean difference (SMD) of tumor volume (the unit is mm^3^) from baseline or before thermal ablation to the last follow-up after therapy (6 studies involved) are summarized in **Figure 3A** with corresponding forest plots. Using the random-effects model, RFA, MWA, and LA all induced a statistically significant reduction in nodule volume after ablation (I² = 64%, τ² = 0.3438, P = 0.02). Pooled SMD is 1.53 (95% Confidence interval(CI): -2.24; -0.82). **Figure 3B** also shows a great reduction rate (the unit is %) of lymph node volume change (I² = 71%, τ² = 0.1439, P < 0.01). We used both the fixed-effects model and the random-effects model to calculate the pooled reduction rate, which was 0.94 (95% CI: 0.92; 0.96) and 0.93 (95% CI: 0.87; 0.97), respectively.

### Changes in the Serum Tg Level After LA, RFA and MWA

**Figure 4:**
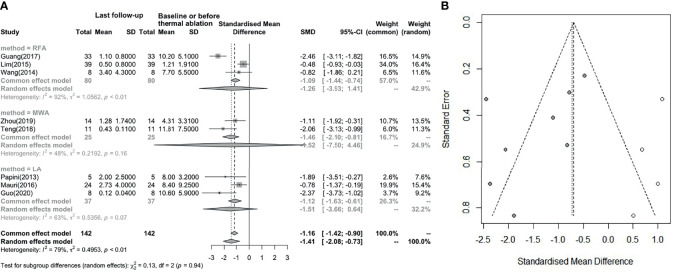
**(A)** Before and after changes in serum Tg level. **(B)** Revised funnel plots of changing in serum Tg level.

**Figure 4A** indicates that the serum Tg level (8 studies involved) showed a statistically significant decrease after thermal ablation (I² = 79%, t^2^ = 0.4953, P < 0.01). The pooled SMD is -1.41 (95%CI: -2.08; -0.73) and no difference between groups. From the funnel diagram in **Figure 4B**, it can be seen that the points are evenly distributed, but there is great heterogeneity, which cannot be avoided.

### Frequencies of Complete Disappearance and Effective Rate After LA, RFA and MWA

**Figure 5:**
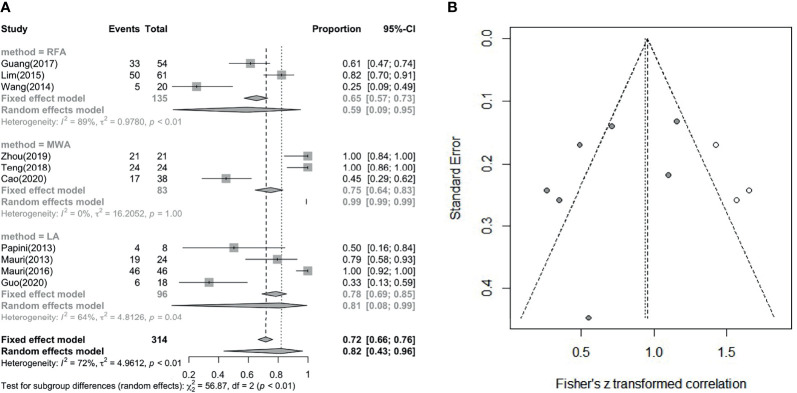
**(A)** Pooled complete disappearance rate. **(B)** Revised funnel plots of complete disappearance rate.

The comparison between frequencies of complete disappearance (7 studies involved) is shown in **Figure 5A** (I² = 72%, τ² = 4.9612, P < 0.01) (correlation coefficient(COR) = 0.72, 95% CI: 0.66; 0.76; COR = 0.82, 95% CI: 0.43; 0.96) and **Figure 5B**. The outcome of subgroup analysis showed complete disappear rate which LA is higher than RFA, while MWA cannot entail in the conclusion because of its statistical significance. Since the number of data points was less than 10, we used Peters’ test to calculate publication bias. After using the trim and filled model for correction, the outcome is presented in the form of forest plots, with low publication bias (**Figure 5B**) (36). Wang (2014) and Mauri (2013) have effective rates of 0.75 and 0.73, and Han did not report this data, but all of the other studies have an effective rate of 100%. So, the overall effective rate is 97.67% (210/215). In the analysis, we can see that the effective rate is very high, while the complete disappearance rate is relatively low.

### Frequencies of Recurrence and Distant Metastasis After LA, RFA and MWA

After resection of the primary tumor, a series of ablation treatments for metastatic lymph nodes was performed. Theoretically, there is still a certain probability of local recurrence and distant metastasis. In these 11 studies (**Table 2**), six had no local recurrence or distant metastasis. Other studies had a small number of local recurrences or distant metastases. However, they were all effectively controlled after further LA, RFA, or MWA treatment. Regarding the ablation time and energy, 7 studies mentioned times ranging from 12 s to 1200 s, and 6 studies mentioned energies ranging from 70 J to 36000 J. Guo (16) thought that the ablation approach should be adjusted for the individual nodes, and we think this may be the reason for the wide variation in ablation time and energy.

**Table 2:** Recurrence rate and distant metastasis situation associated with ablated time and energy.

Author	Local recurrence	Distant metastasis	Ablated time (s)	Ablated energy (J)
Guo (2020) (16)	0	0	Depend on individual nodes	Depend on individual nodes
Teng (2018) (12)	0	0	75.63 ± 45.44 (12-174)	1512.5 ± 908.8 (240-3480)
Zhou (2019) (11)	0	0	93.9 ± 56.9 (30-190)	NA
Cao (2020) (18)	0	0	NA	NA
Han (2020)* (19)	0	0	206.55 ± 193.59	NA
Guang (2017)* (9)	0	1	140.7 ± 88.4 (24-447)	426.7 ± 279.8 (70-1320)
Wang (2014)* (10)	0	1	162 (30-360)	NA
Papini (2013) (14)	0	2	NA	942 ± 342 (573-1574)
Mauri (2013) (13)	5	3	NA	1200-4200
Mauri (2016) (15)	4	3	300-600	1200-4200
Lim (2015) (22)	0	0	243.5 ± 264.7 (33-1200)	3936.4 ± 5960.9 (370-36000)

Han (2020)*: Patients with local recurrence or distant metastasis during follow-up were excluded.

Guang (2017)*:During the 12-month follow-up, distant metastasis was found in one patient, and was successfully ablated later.

Wang (2014)*: Two new cervical recurrent lymph nodes occurred in one case, which was finally ablated.

### Frequencies of Complications From LA, RFA and MWA

**Figure 6:**
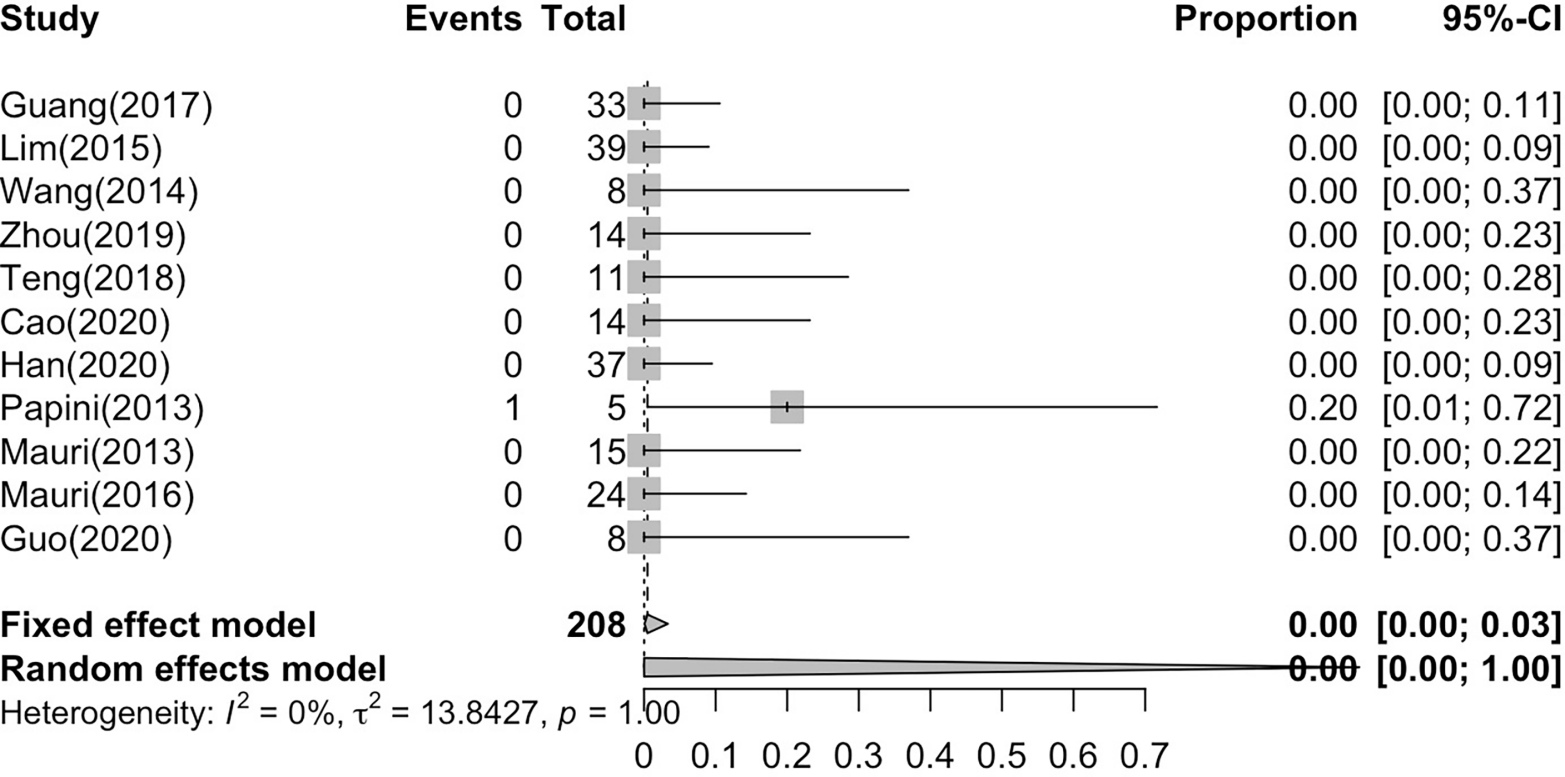
Pooled major complication rate.

**Figure 7:**
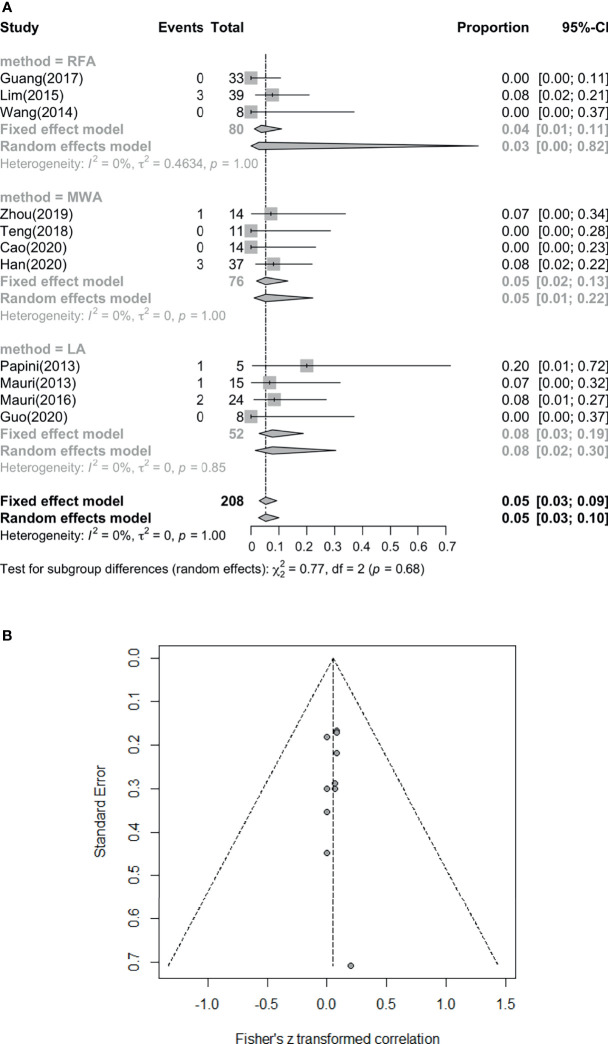
**(A)** Pooled overall complication rate. **(B)** Funnel plots of overall complication rate.

The major complication rate and overall complication rate are compared in **Figure 6** and **Figure 7A**, in which we can see I² = 0%, P = 1.00, showing that there is no statistically significant difference (P > 0.05), although the heterogeneity is low (**Figure 7B**). Therefore, there was no difference between the two complication ratios. On the other hand, the incidence of the major complication rate was 1/208 (0.48%), and the overall complication rate was 11/208 (5.29%). The incidence of complications remains low, and the safety of the treatment is guaranteed and there is no subgroup difference.

## Discussion

This meta-analysis showed that US-guided thermal ablation is a valid therapeutic method for LNM of PTC. RFA, MWA and LA all resulted in a significant reduction in metastatic lymph nodes (P = 0.02), and the reduction rate was 93%-94% (P < 0.01). In addition, during the follow-up process ranging from 6 to 24 months, the patient’s diseased lymph node size was effectively controlled, and the serum Tg level was also effectively improved (P < 0.01), this is similar with a latest meta-analysis (37). However, we performed a pooled summary analysis of the main complications and overall complications, and a detailed subgroup analysis of the complete disappearance rate was performed. In the pooled analyses of the complete disappearance rate, the effective rate, and the major and overall complications (including major and minor complications), we found that there were few complications after thermal ablation (P = 1 not statistically significant). Almost all complications were minor and disappeared over time. Furthermore, almost all subgroup analysis showed no significant difference among thermal ablation methods, only except for LA is better than RFA in complete disappearance rate. In addition, we carried out the bias correction of the funnel plot for each forest plot, so that the bias of the article cases can be more clearly reflected. We use statistical analysis to specify each P value, and the results are more reliable and have certain clinical significance.

In some other documents, these three thermal ablation treatments were compared with surgical treatments for different thyroid diseases, and they all showed effectiveness and safety at different levels, including lower complication rates and only minor side effects. Therefore, they are considered to be better alternatives to surgical treatment (6–8, 38–40). In all of the analyses performed, statistically significant data showed the effectiveness of all three thermal ablation methods. Although there were some metastases and complications in some literature reports, all of them returned to normal after intervention during the follow-up process, so they were classified as minor complications. In addition, in this meta-analysis, no case reported life-threatening or irreversible complications, so thermal ablation is suitable for treating LNM of PTC. These findings are consistent with previous research findings, many literatures have shown the effectiveness and safety of the three thermal ablation methods for various types of thyroid nodules and lymph nodes (41, 42). In the ablation of thyroid nodules, some literatures have compared RFA with LA and found that RFA is faster than LA which led to a conclusion that regeneration is less but requires higher energy (43, 44). There is also literature comparing RFA and MWA, suggesting that RFA may have a better long-term effect in reducing nodule volume (45).

Based on the results of this systematic review and meta-analysis, we still feel very encouraging and confident for clinicians to perform promising and innovative treatment methods for patients who cannot tolerate surgery or explicitly refuse surgery. This ought to improve patient outcomes and allow them to avoid the physical and mental trauma caused by multiple surgeries. During the execution of thermal ablation therapy, patients received local anesthesia, and injected an isolation fluid around the lymph nodes to prevent thermal injury to important anatomy such as peripheral blood vessels and nerves (16, 18). This also ensures the effectiveness and safety of the treatment. In addition, percutaneous TA treatment has good feasibility and reproducibility, it can be performed many times until the desired therapeutic effect is achieved, and without increased technical difficulties due to the previous treatments. This is undoubtedly a gratifying gospel for patients with multiple recurrence of lymph nodes.

When summarizing the heterogeneity of all studies (46, 47), we found that the heterogeneity of the major complication rate and overall complication rate were 0 (but not statistically significant), but the rest of the outcomes showed the characteristics of medium-to-high heterogeneity (I^2^ > 50%). Although we used both fixed-effects and random-effects models, subgroup analysis should also be used to obtain each characteristic of the three thermal treatments. However, since there are relatively few studies reporting on each modality at present, the cases we have obtained are relatively limited. We hope that we can obtain more comprehensive information on thermal ablation in the future.

The present meta-analysis also has several limitations. First, the sample size of the studies was limited, and most were retrospective, which may have affected the accuracy of the results. Second, after summarizing the data of these three thermal ablation methods (RFA, MWA, LA), we found that doctors use different ablation strategies for different patients (including the time, frequency, and energy of ablation). Therefore, a multicenter and large sample study is needed to determine a more standardized and unified ablation strategy. In addition, our research regards the situation of last control as the final result, so detailed analysis during long-term follow-up was not conducted. For clinical guidance, we can conclude that the use of thermal ablation has a good therapeutic effect on LNM of PTC. It can reduce the volume of the lymph nodes to a large extent, but it requires additional long-term follow-up to pay attention to the disease factors.

In general, by analyzing the three thermal ablation treatments, we found that thermal ablation is safe and efficacious and will be an option for treatment with great potential for LNM in the future. However, its limitations include a lack of standardized procedures and the requirement for experienced doctors, and longer-term follow-up periods are necessary.

## Data Availability Statement

The original contributions presented in the study are included in the article/supplementary material. Further inquiries can be directed to the corresponding authors.

## Author Contributions

Guarantors of integrity of entire study: ZD, JC, XC, and LS. Literature research: ZD, JC, XC, and LS. Study concepts/study design: all authors. Contributed to acquisition of data: ZD and JC. Contributed reagents/materials/analysis tools: ZD and JC. Manuscript drafting or manuscript revision: all authors. Statistical analysis: all authors. All authors contributed to the article and approved the submitted version.

## Conflict of Interest

The authors declare that the research was conducted in the absence of any commercial or financial relationships that could be construed as a potential conflict of interest.

## Publisher’s Note

All claims expressed in this article are solely those of the authors and do not necessarily represent those of their affiliated organizations, or those of the publisher, the editors and the reviewers. Any product that may be evaluated in this article, or claim that may be made by its manufacturer, is not guaranteed or endorsed by the publisher.
